# Supplementary material for: Resolution of R-loops by INO80 promotes DNA replication and maintains cancer cell proliferation and viability
Source: Nat Commun. 2020 Sep 10;11:4534. doi: 10.1038/s41467-020-18306-x (PMC7484789; doi:10.1038/s41467-020-18306-x)
Supplement: Supplementary file 1 — Supplementary Information [file 41467_2020_18306_MOESM1_ESM.pdf]

Supplementary Information

12 Supplementary Figures with Legends.

1 Table.

4 Movies.

Supplementary Figure 1

a.

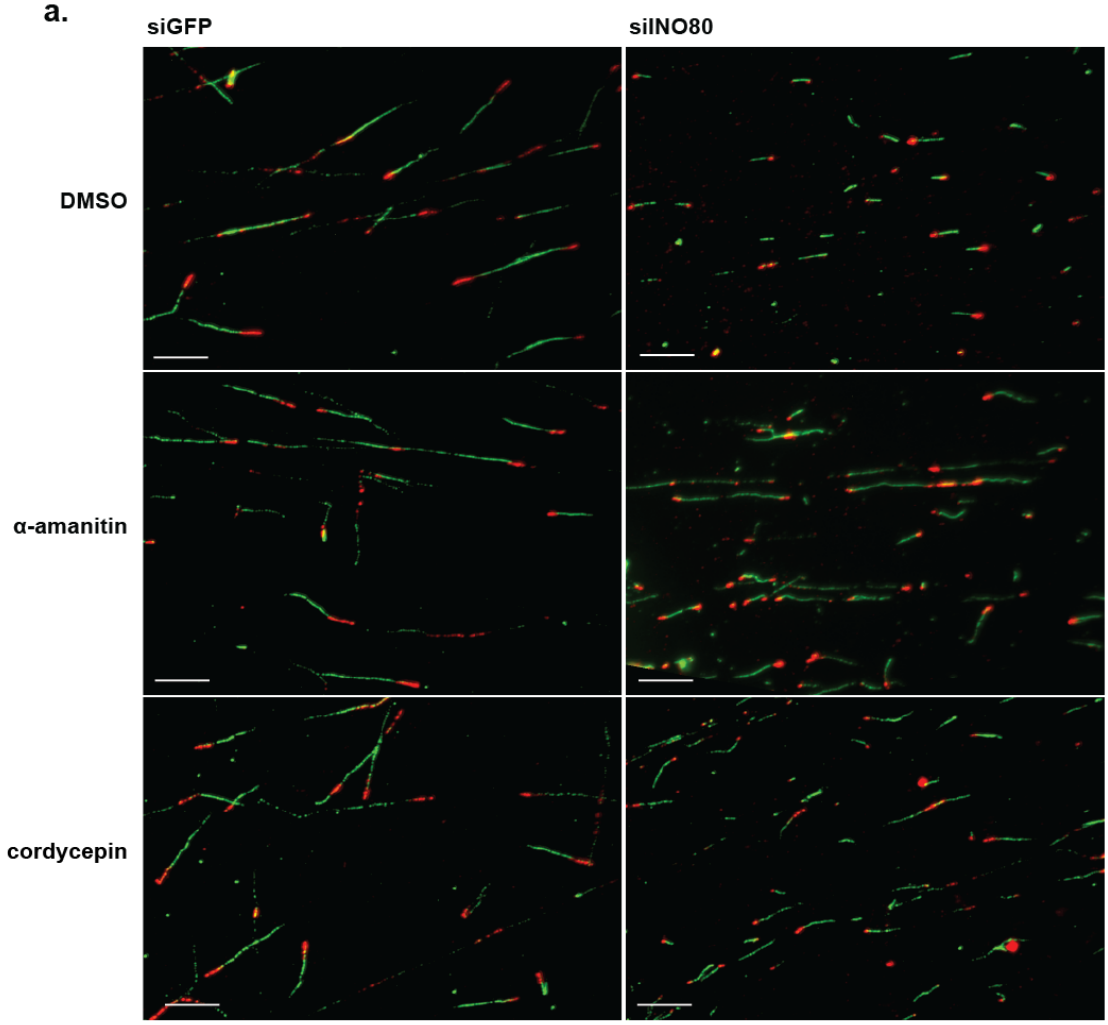

b.

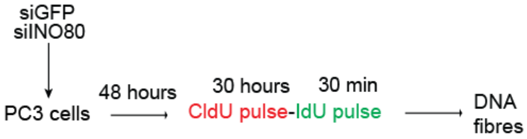

c.

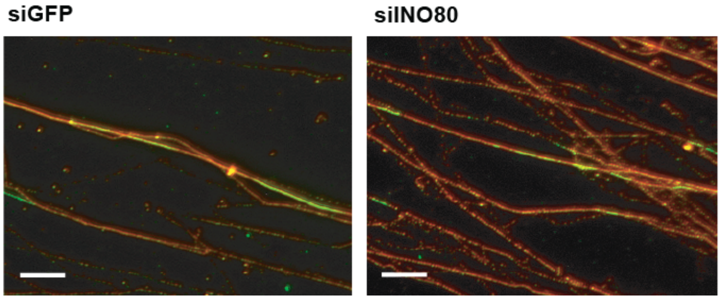

Supplementary Fig. 1 Representative large images of spread fibres. **(a)** Representative large images of spread fibres from each condition in Figure 1b. Scalebar 10μm. Similar results were obtained in 5 independent repeats with α-amanitin and 2 independent repeats with cordycepin. **(b)** Labelling scheme to control and INO80-depleted cells to check fibre integrity. First label was added for 30h to uniformly label genomic DNA. **(c)** Representative images of each condition. Scale bar 5μm. Similar results were obtained in 3 independent experiments.

Supplementary Figure 2

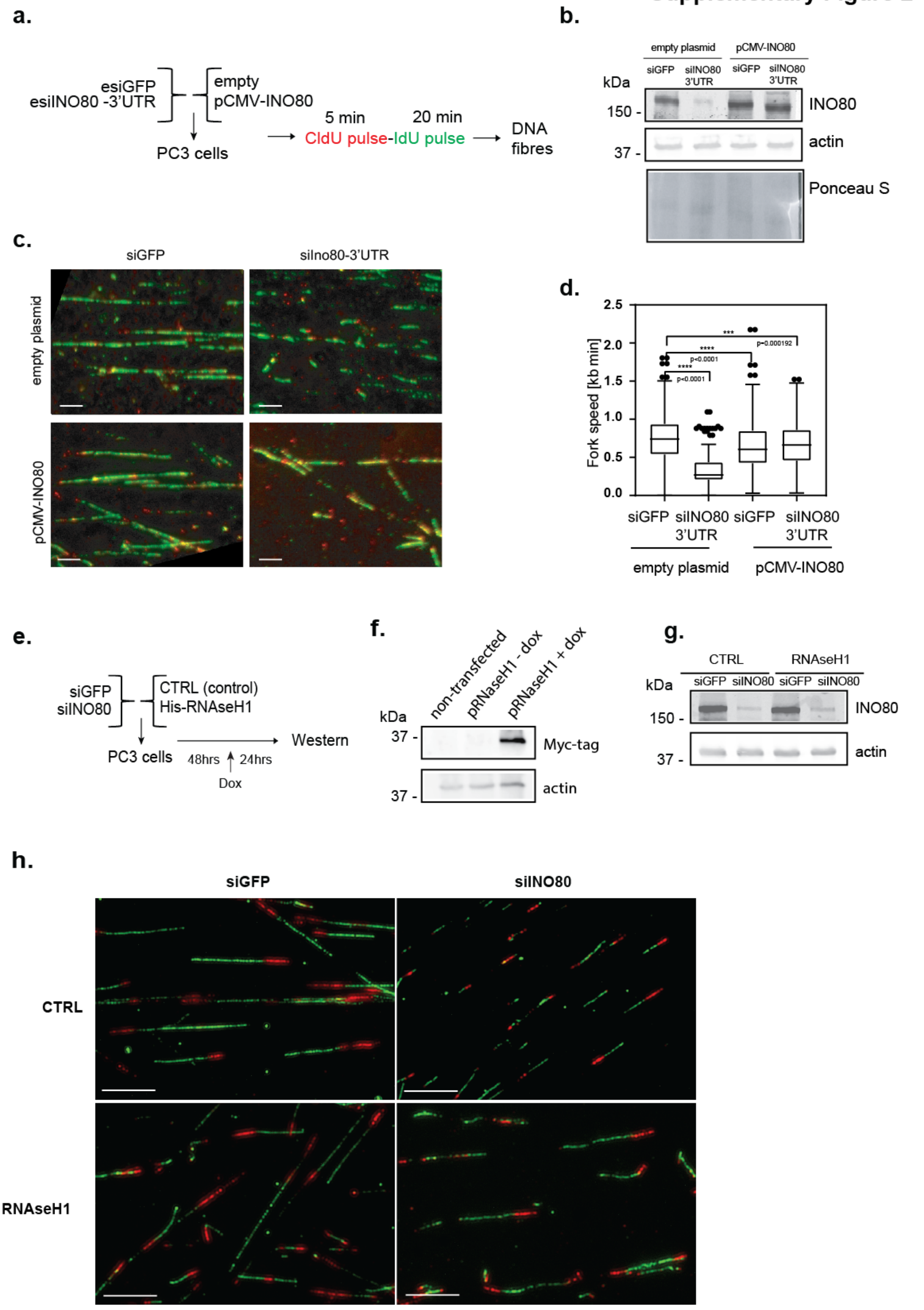

Supplementary Fig. 2. Replication defect in INO80-depleted cells is rescued by expression of knock-down resistant INO80 and RNAse H1 overexpression. (a) Experimental scheme. PC3 cells were cotransfected with an empty vector and either esiRNA against GFP or INO80-3'UTR and with a plasmid expressing INO80 and esiRNA against GFP or against INO80-3'UTR. Seventy two hours later cells were subjected to DNA fibre labelling. **(b)** Western analysis of Ino80 levels in cells co-transfected with an empty vector and either esiRNA against GFP (lane 1) or INO80 3'-UTR (lane 2) and ones co-transfected with INO80 -expressing plasmid (pCMV- INO80) and esiRNA against GFP (lane 3) or INO80 3'-UTR (lane 4). Similar results were obtained in two independent experiments. **(c)** Cells transfected as in (a) were subjected to DNA fibre labelling. Representative images are shown. Scale bar 5μm. Similar results were obtained in three experiments. **(d)** Distribution of fork speed rates (kilobase/min) in cells co-transfected with siGFP and siINO80-3'UTR together with empty plasmid or Ino80-overexpressing one. At least 250 fibres were measured per condition. ****p-value < 0.0001; ***p-value < 0.001, (two-tailed unpaired Student's t-test). In Kruskal-Wallis test p-value was < 0.0001. **(e)** Schematic representation of the experimental setup. PC3 cells were co-transfected with esiRNAs against either GFP (siGFP) or INO80 (siINO80) along with either a control (CTRL) or with a plasmid expressing RNAseH1 tagged with His and Myc-tags under the control of doxycycline-inducible CMV promoter. Two days later RNAse H1 expression was induced with 1 μg/ml Doxycycline for 24 hours. **(f)** RNAseH1 induction was determined in induced, uninduced and untransfected cells by Western with an anti- Myc-tag antibody. Similar results were obtained in 2 independent repeats. **(g)** Western blot with an anti-Ino80 antibody of total extracts from PC3 cells co-transfected with esiRNAs against GFP or INO80 together with either control or RNAseH1 overexpressing plasmids, transfected and induced as in (e). Similar results were obtained in 2 independent experiments. **(h)** Representative large images of spread fibres from each condition in Figure 1e. Similar results were obtained in 4 independent experiments. Scale bar 10 μm. Source data are provided as a Source Data file.

Supplementary Figure 3

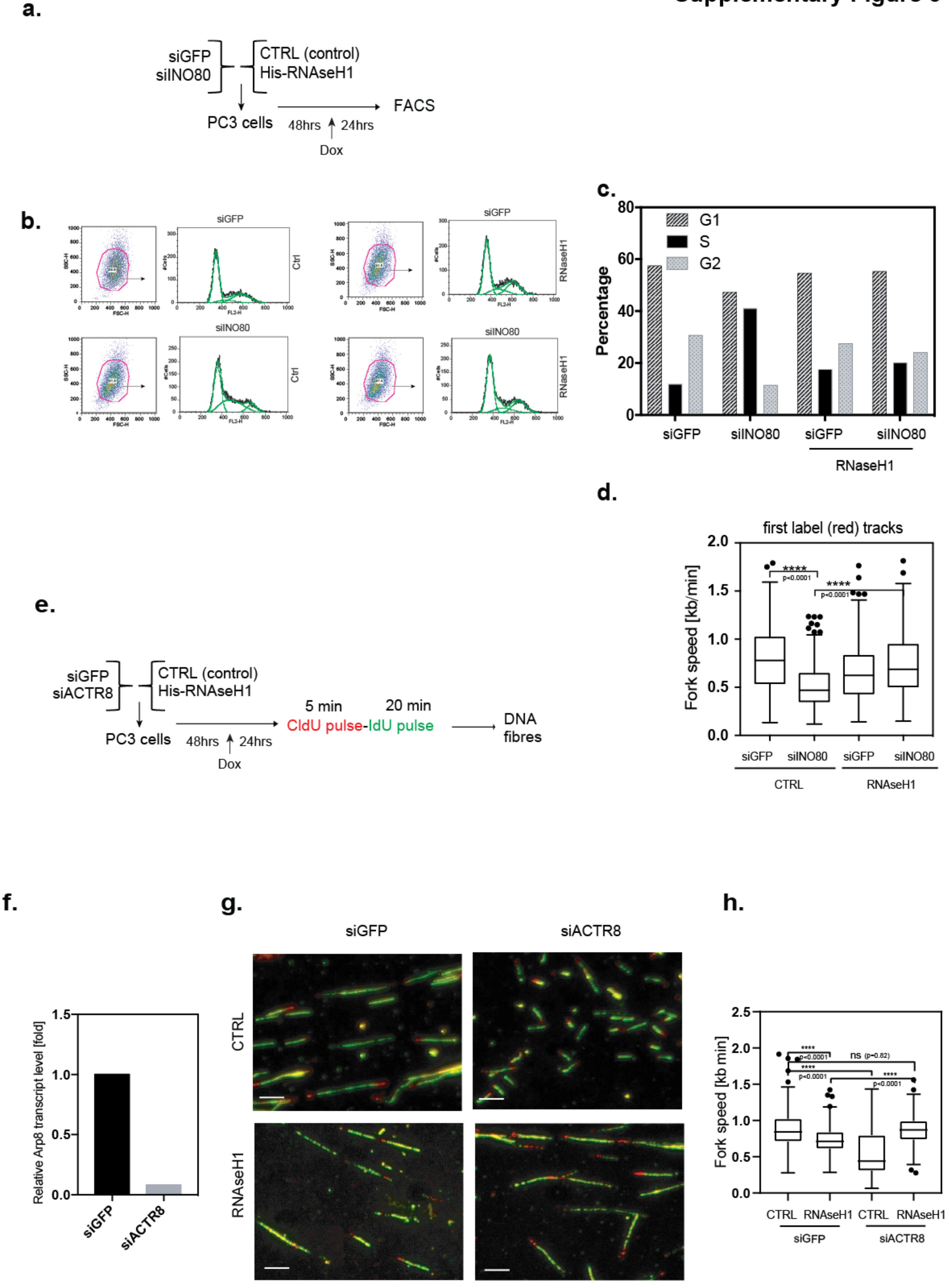

Supplementary Fig. 3. RNAse H1 overexpression rescues the slow DNA replication defect of INO80-depleted and ACTR8-depleted cells. (a) Schematic representation of the experimental setup. PC3 cells were co-transfected with esiRNAs against either GFP (siGFP) or INO80 (siINO80) along with either a control (CTRL) or RNAseH1-overexpressing (RNAseH1) vector. Two days later RNAse H1 expression was induced by doxycycline for 24 hours. **(b)** Cell cycle profiles of cells transfected and induced as in (a), stained with propidium iodide and subjected to flow cytometry. Green lines indicate data fitted using the Dean-Jett-Fox model. Gating is shown to the left of the cell cycle plot. **(c)** Cell cycle distribution of cells from (b). Ten thousand cells were counted per sample. The experiment was repeated twice. Percentages are calculated by FlowJo from the fitted model[1]. **(d)** Replication fork rates estimated measuring the first (red) label of cells processed as in Fig. 1d and subjected to DNA fibre labelling. Data is from 3 experiments with at least 100 tracks per sample scored in each experiment. In Kruskal-Wallis test p-value was < 0.0001. Data is presented as Tukey boxplot. **(e)** Schematic representation of the experimental setup used. PC3 cells were co-transfected with esiRNAs against either GFP (siGFP) or ACTR8 (siACTR8) along with either a control (CTRL) or RNAseH1-overexpressing (RNAseH1) vector. Two days later RNAse H1 expression was induced by doxycycline for 24 hours. Cells were labelled with CldU for 5 minutes followed by IdU pulse for 20 minutes and subjected to DNA fibre labelling analysis. **(f)** Quantitative RT-PCR to assess mRNA level of ACTR8 in 3 days after transfection of PC3 cells with the indicated esiRNA. Expression was scored in three independent samples per each condition. **(g)** Representative images of spread fibres from control (siGFP) or ACTR8-depleted (siACTR8) cells with (RNAseH1) or without (CTRL) RNHase H1 overexpression. Similar results were obtained in 2 independent experiments. Scale bar 5μm. **(h)** Distribution of fork speed rates (kilobase/min) in siGFP and ACTR8-deficient cells transfected with control or RNAseH1 overexpression plasmids. At least 250 fibres were measured per condition obtained in two independent experiments. ****p-value < 0.0001, (two-tailed unpaired Student's t-

test). In Kruskal-Wallis test p-value was < 0.0001. Data is presented as Tukey boxplot.

Supplementary Figure 4

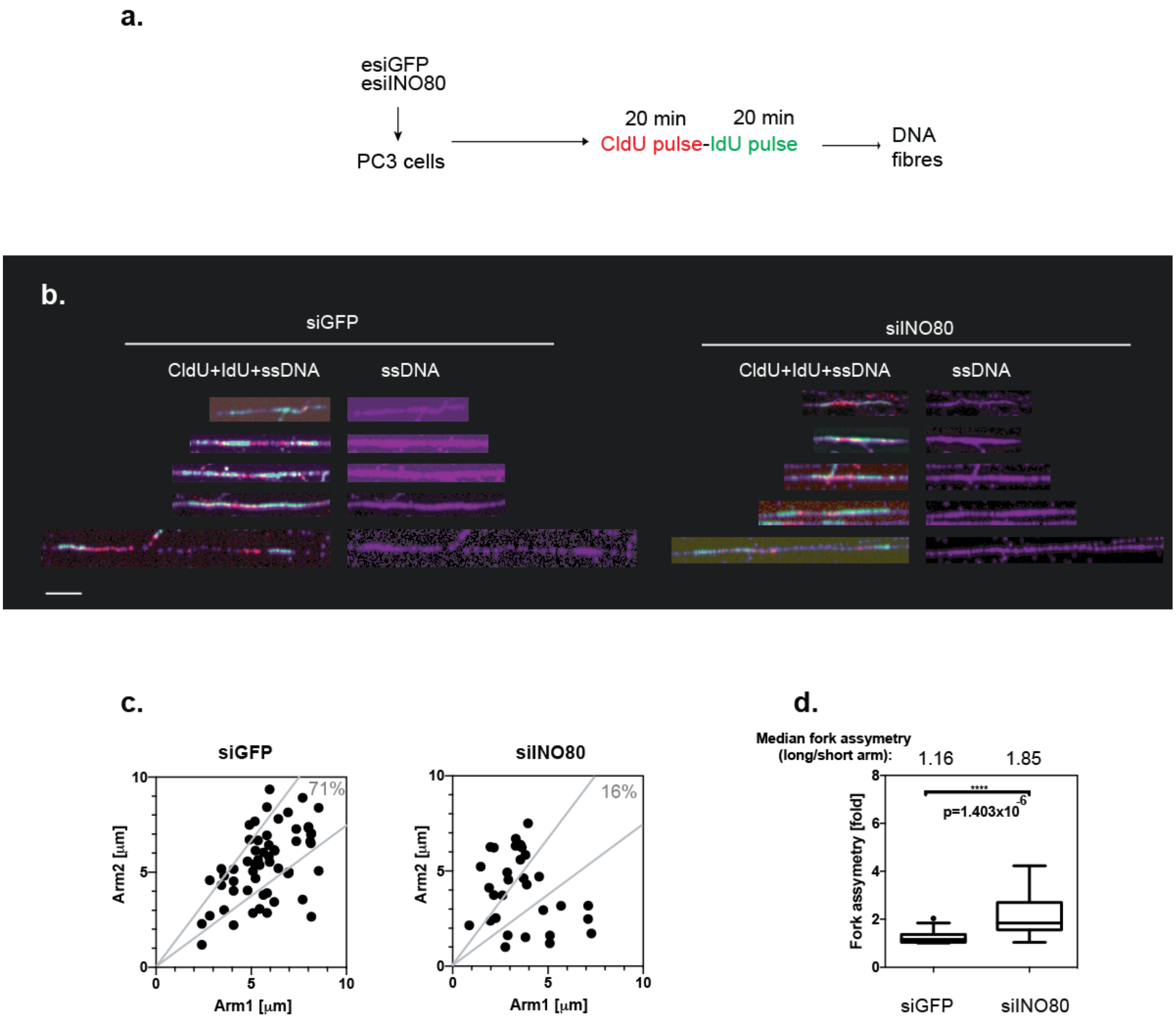

Supplementary Fig. 4 Fork asymmetry in cells depleted of Ino80. (a) Schematic representation of the experimental setup used. PC3 cells were transfected with esiRNAs against either GFP (siGFP) or INO80 (siINO80). Cells were labelled with CldU for 20 minutes (red) followed by IdU pulse for 20 minutes and subjected to DNA fibre labelling analysis. To control for fibre integrity an antibody to stain single stranded DNA (ssDNA) was used. **(b)** Representative pairs of sister replication forks were assembled from different fields. Three-color staining images (CldU, IdU and ssDNA) are shown along with ssDNA image of the same fibre to the right. Similar results were obtained in 2 independent experiments. Scale bar 5µm. **(c)** Scatter plots of the distances covered by right-moving and left-moving sister forks at unbroken DNA fibres during the CldU pulse in INO80-proficient or deficient cells. The central areas, delimited with grey lines, contain sister forks with less than a 25% length difference. The percentage of symmetric forks is indicated. **(d)** Relative fork asymmetry. Fork asymmetry is expressed as the ratio of the longer arm to the shorter one for each pair of sister replication forks during the ldU (green) pulse, Numbers above boxes indicate the median of the ratio of the longer to shorter arm at unbroken DNA fibres. Data from 30-35 clearly intact DNA fibres as identified by ssDNA staining were measured in each condition in one experiment. ****p-value < 0.0001; (two-tailed unpaired Student's t- test). Tukey boxplot is shown.

Supplementary Figure 5

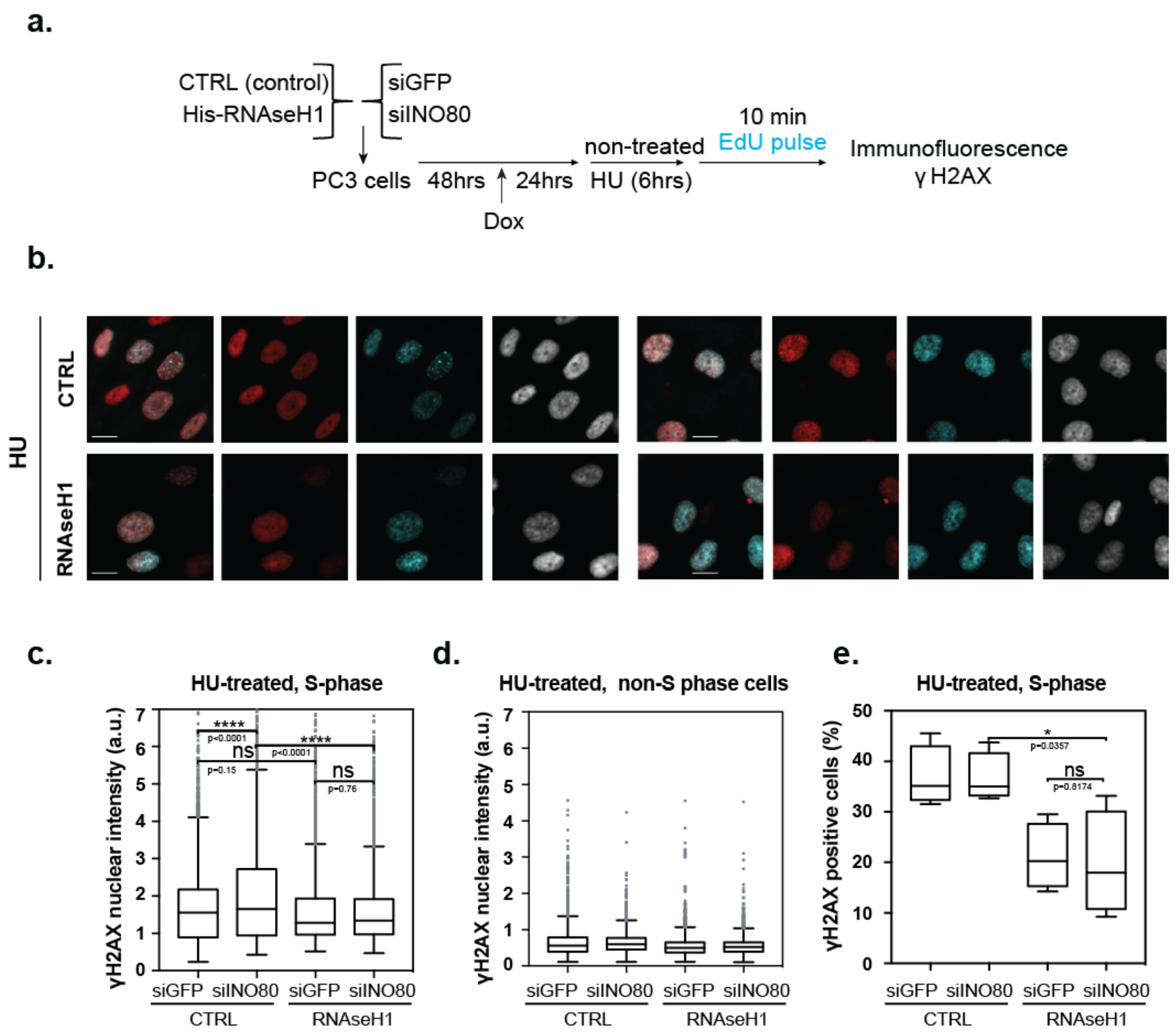

Supplementary Fig. 5 (a) Schematic representation of the experimental setup used. PC3 cells were co-transfected with esiRNAs against either GFP (siGFP) or INO80 (siINO80) along with either a control (CTRL) or RNAseH1-overexpressing (RNAseH1) vector. Two days later RNAseH1 overexpression was induced by doxycycline for 24 hours. Cells were treated with 0.5 mM HU for 6h or left untreated as control. To distinguish cells in S-phase, cells were labelled with 25 µM EdU, fixed and stained with an antibody against γH2AX and "clicked" with Alexa Fluor 488 azide. **(b)** Representative images of cells treated as in (a). Scale bar – 10µm. Similar results were obtained in 3 independent experiments. **(c)** Distribution of nuclear γH2AX staining intensities in S-phase cells treated with HU. Data is from 3 independent experiments following normalization (as in Fig. 3d). ****p-value < 0.0001, * p-value < 0.05, ns – nonsignificant (two-tailed unpaired Student's t-test). Data is presented as Tukey boxplot. **(d)** Distribution of γH2AX staining intensities in non-S-phase cells. 3 independent experiments with at least 500 nuclei counted per condition in each experiment. Data is presented as Tukey boxplot.. **(e)** Mean percentages of γH2AX-positive population in cells treated as in (a). Data is from 4 independent experiments; * p-value < 0.05, ns – nonsignificant (two-tailed unpaired Student's t-test). Data is presented as Tukey boxplot.

Supplementary Figure 6

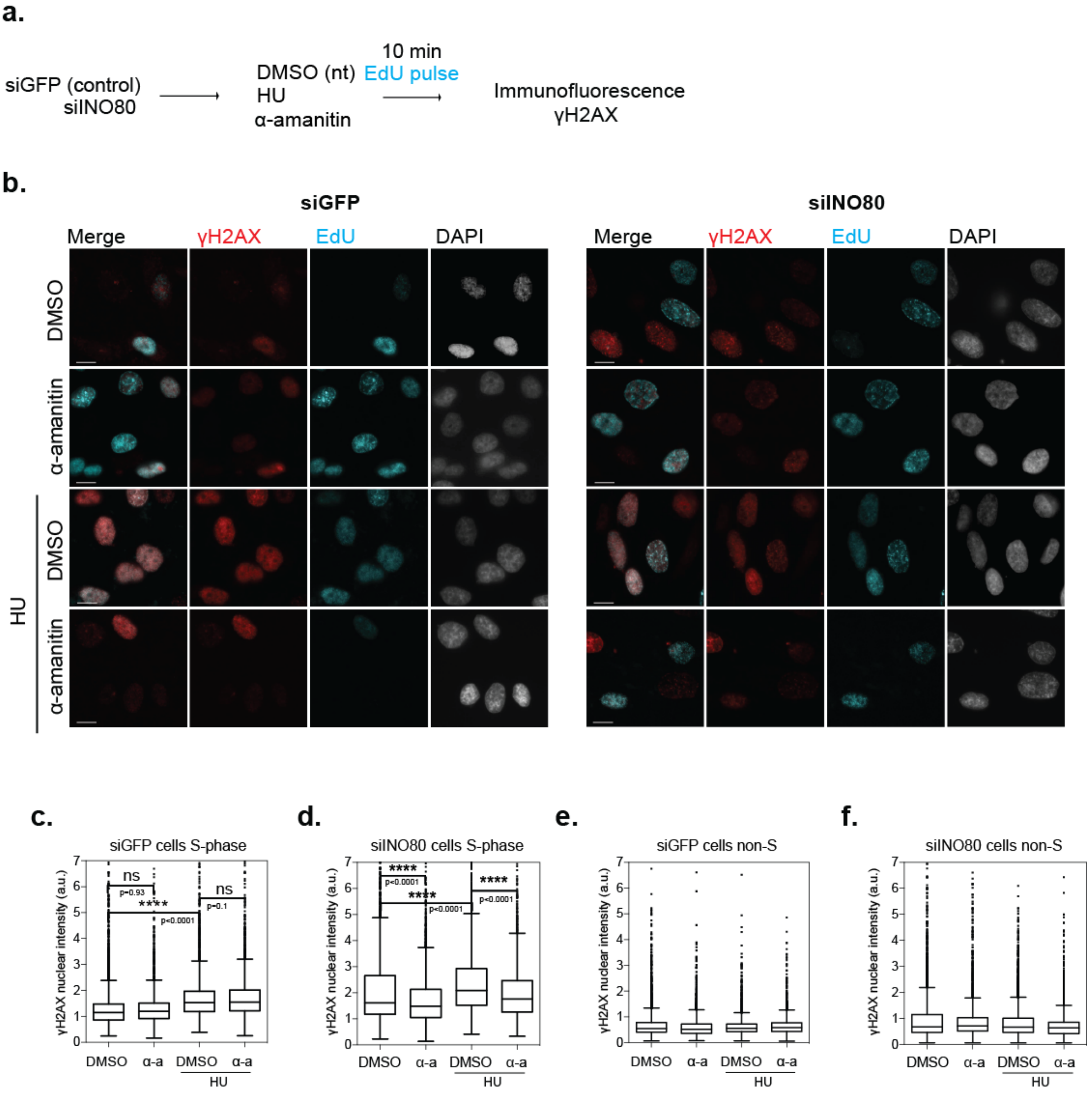

Supplementary Fig. 6. Replication stress-induced DNA damage in INO80-deficient cells is caused by co-transcriptional R-loops.

(a) Schematic representation of the experimental approach. PC3 cells were transfected with esiRNAs against either GFP or INO80. Cells were treated with transcription inhibitor α-amanitin (α-a) and/or hydroxyurea (HU) or both for 6h as indicated (α-amanitin and HU being added simultaneously). Cells were labelled with 25 μM EdU to distinguish the ones in S-phase. Cells were fixed and stained with an antibody against γH2AX and "clicked" with Alexa fluor 488 azide. **(b)** Images of cells treated with transcription inhibitors, HU or both, and controls. Scale bar – 10μm. Similar results were obtained in 3 independent experiments. **(c)** Distribution of γH2AX nuclear intensities from siGFP S-phase cells as in (a). Data is from 3 independent experiments after normalization (as in Fig. 3d), with at least 500 cells analyzed per condition in each experiment; ****p-value < 0.0001, ns – nonsignificant (two-tailed unpaired Student's t-test). Data is presented as Tukey boxplot. **(d)** Distribution of γH2AX nuclear intensities from siINO80 S-phase cells. Data is from 3 independent experiments after normalization, at least 500 cells were analyzed per condition in each experiment; ****p-value < 0.0001, (two-tailed unpaired Student's t-test). Data is presented as Tukey boxplot. **(e)** Distribution of nuclear γH2AX staining intensities in siGFP non-S phase cells. Data was obtained in 3 independent experiments with at least 500 nuclei analysed per condition per experiment. Data is presented as Tukey boxplot. **(f)** Distribution of nuclear γH2AX staining intensities in siINO80 non-S phase cells. Data is from 3 independent experiments with at least 500 nuclei analyzed per condition per experiment. Data is presented as Tukey boxplot.

Supplementary Figure 7

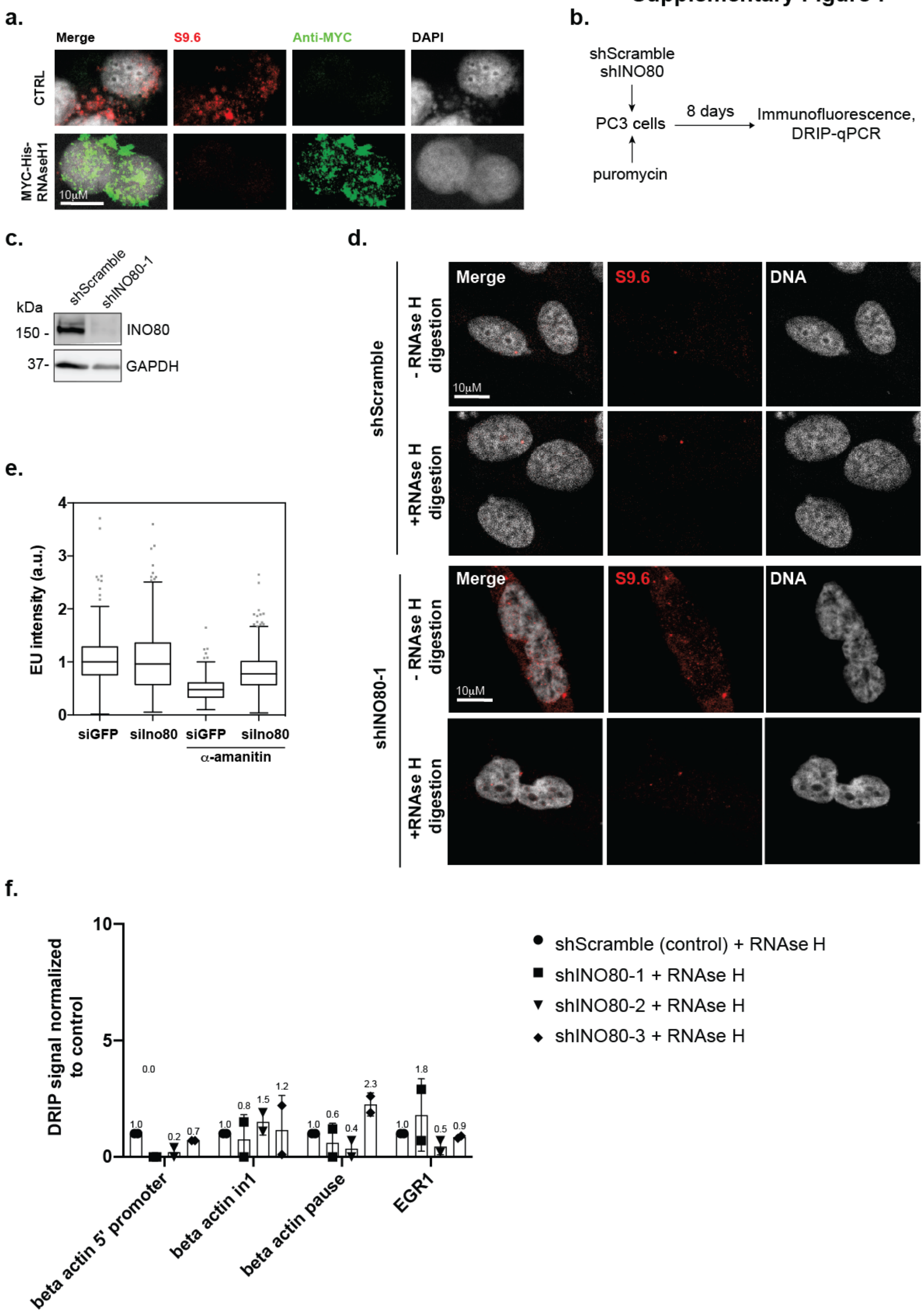

Supplementary Fig. 7. Transcription and R-loop analyses.

(a) Overexpression of RNAse H1 induces reduction in R-loops. Representative non-deconvolved immunofluorescence image of PC3 cells which were transfected with plasmid control (CTRL) or plasmid overexpressing RNAse H1 for 24h, fixed and immunostained with the S.9.6 antibody against R-loops and myc antibody against RNase H1-myc. Scale bar is 10μM **(b)** Schematic representation of the experimental setup used for depletion of INO80 by shRNA. **(c)** Immunoblot of PC3 cells transduced with control shRNA (ShScramble) RNA and shRNA targeting INO80 (shINO80-1). **(d)** Representative deconvolved immunofluorescence image of control (shScramble) and INO80-depleted (shINO80-1) PC3 cells following digestion with (+) or without (-) recombinant RNAse H. Scale bar is 10μm **(e)** Control (siGFP) or INO80-depleted (siINO80) cells were either treated for 1 h with 2 μg/ml α-amanitin or DMSO. Cells were then labelled with 1mM ethenyluridine (EU) for 3 h (in the presence of α-amanitin), fixed and stained with Alexa Fluor 488 azide. Tukey boxplots represent the distribution of EU intensity. Data is from two independent experiments, at least 1000 cells were analyzed per condition. ****p-value < 0.0001, (two- tailed unpaired Student's t-test); ns, non-significant. **(f)** DRIP-qPCR was performed on the indicated shRNA-treated samples using the S9.6 antibody after digestion of the purified genomic DNA with recombinant RNAse H. Values for each region tested were normalized over shControl after correction for input DNA levels. Data from three independent biological replicates are presented as mean values +/- SD error bars. Source data are provided as a Source Data file.

Supplementary Figure 8

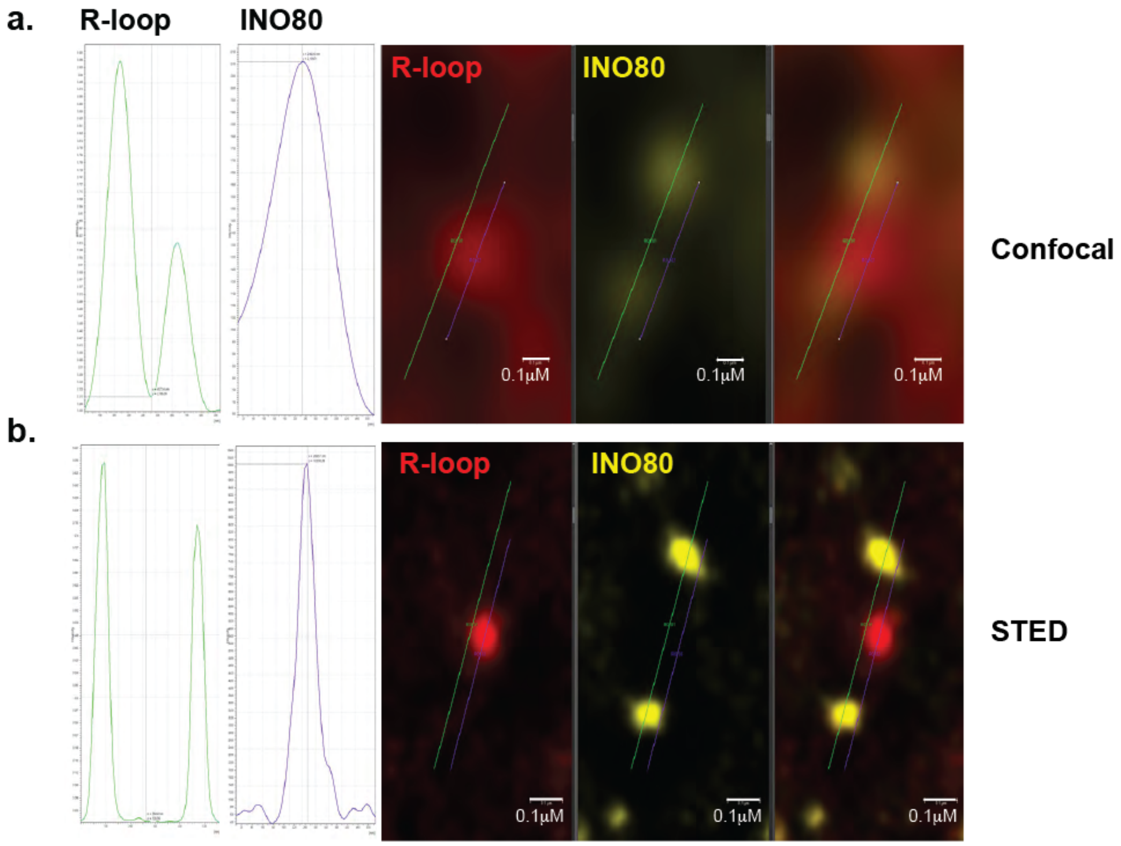

Supplementary Fig. 8. Confocal and STED comparison.

(a) Confocal image of immunostained image of INO80 and S9.6. Fluorescence intensity plots for R-loops (green) and INO80 (purple) indicate that the fluorescent signals overlap, suggesting co-localization at this resolution. **(b)** STED image of immunostained image of INO80 and S9.6 from panel (a). Fluorescence intensity plots for R-loops (green) and INO80 (purple) demonstrate that the peaks for each channel are separate and distinct, with no overlap, therefore no co-localization. Scale bars are 0.1μm.

Supplementary Figure 9

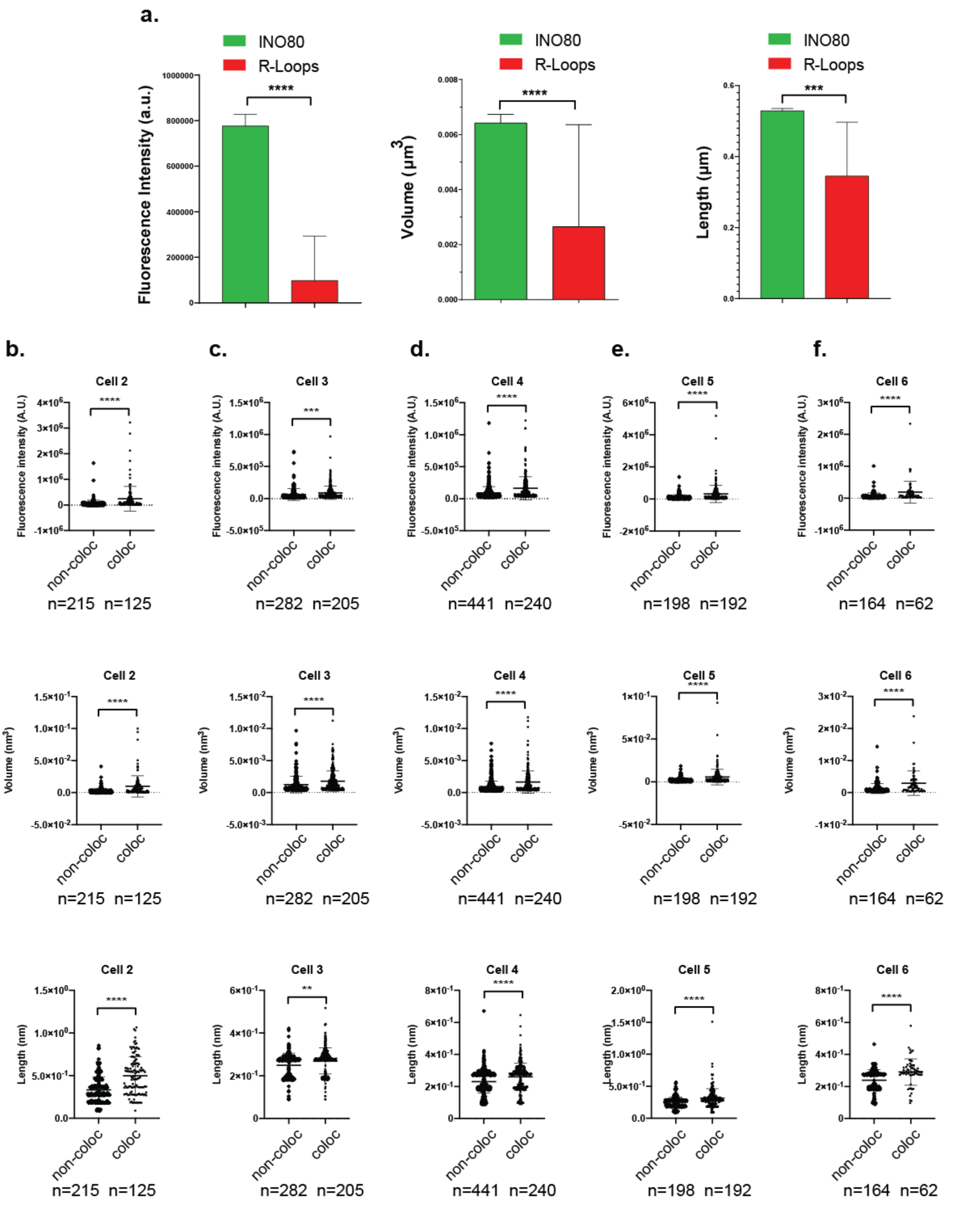

Supplementary Fig. 9. Comparative analysis of the properties of INO80 and R-loop volumes in STED nanoscopy.

(a) INO80 under STED is significantly different in sum of intensity (left panel), Voxel volume (middle panel) and length to R-loops (righ panel) within the same single cell. **(b-f)** Single cell analyses of sum of R-loop intensity (top panels) R-loop voxel volume (middle panels), and length of R-Loops (bottom panels) which co-localize or do not co-localize with INO80 in single cells 2-5. ****p-value < 0.0001; ***p-value < 0.001; **p-value < 0.01 ((adjusted p-values; one way ANOVA). n= number of R-loops per independent cell co-localizing or not colocalizing with INO80. Data are presented as mean values +/- Standard deviation (SD).

Supplementary Figure 10

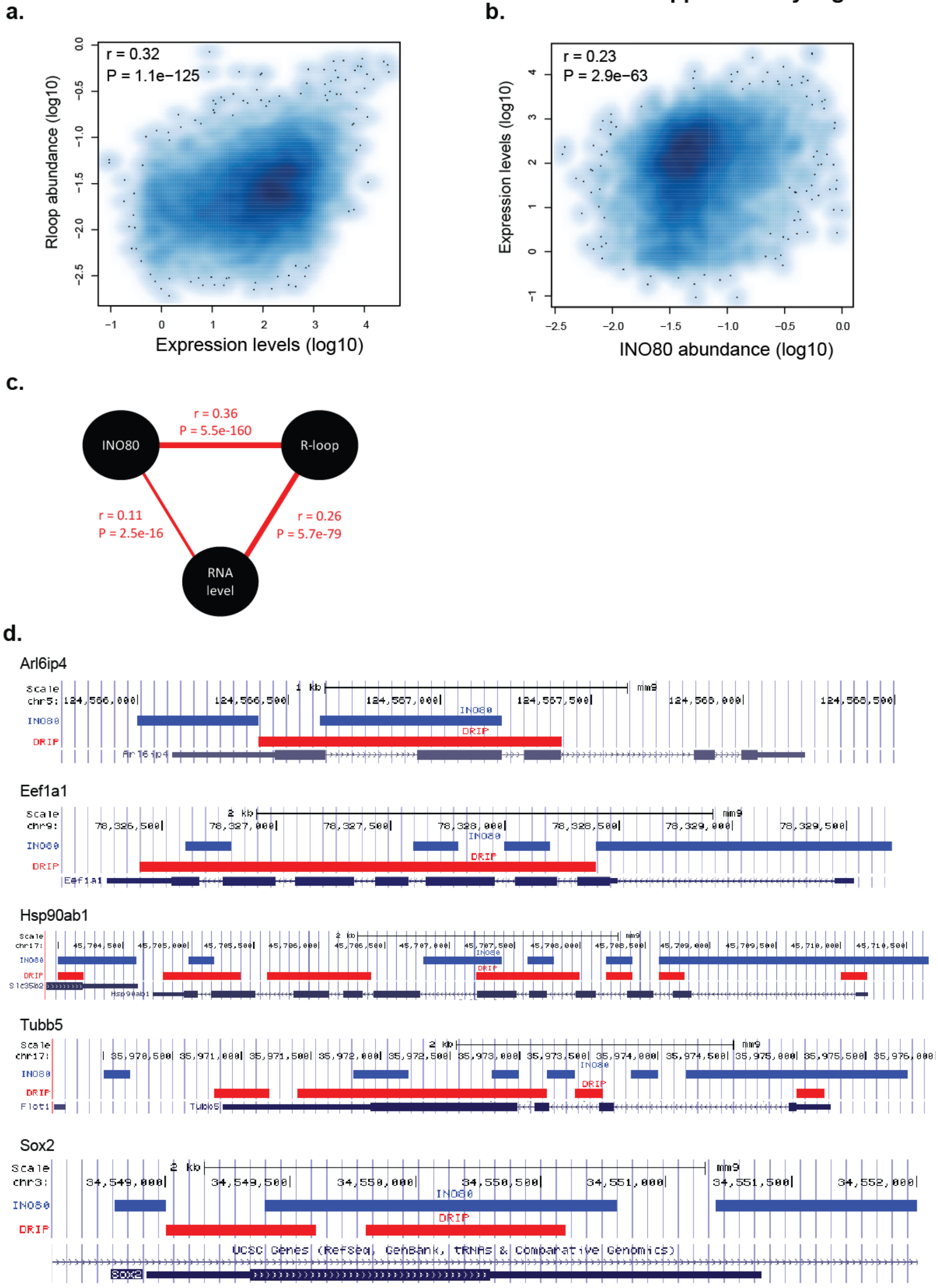

Supplementary Fig. 10. Genome-wide association of INO80 and R-loops.

(a) Smoothed scatterplot showing the pairwise correlation between R-loop and RNA abundances at gene bodies in mESCs. **(b)** Smoothed scatterplot showing the pairwise correlation between RNA levels and INO80 abundances at gene bodies in mESCs. **(c)** Summary network showing partial correlations among INO80, R-loop and RNA levels. The correlation between INO80 and R-loop abundance differs from panel (a) in Figure 6 as it has been adjusted by the correlation of both with RNA levels. **(d)** Genomic enrichment profile of INO80 and R-loops (DRIP) signals across the indicated genes in mouse ESCs. Analysis to visualise the location of INO80 ChIP-seq and DRIP-seq peaks was conducted as in Figure 6b.

Supplementary Figure 11

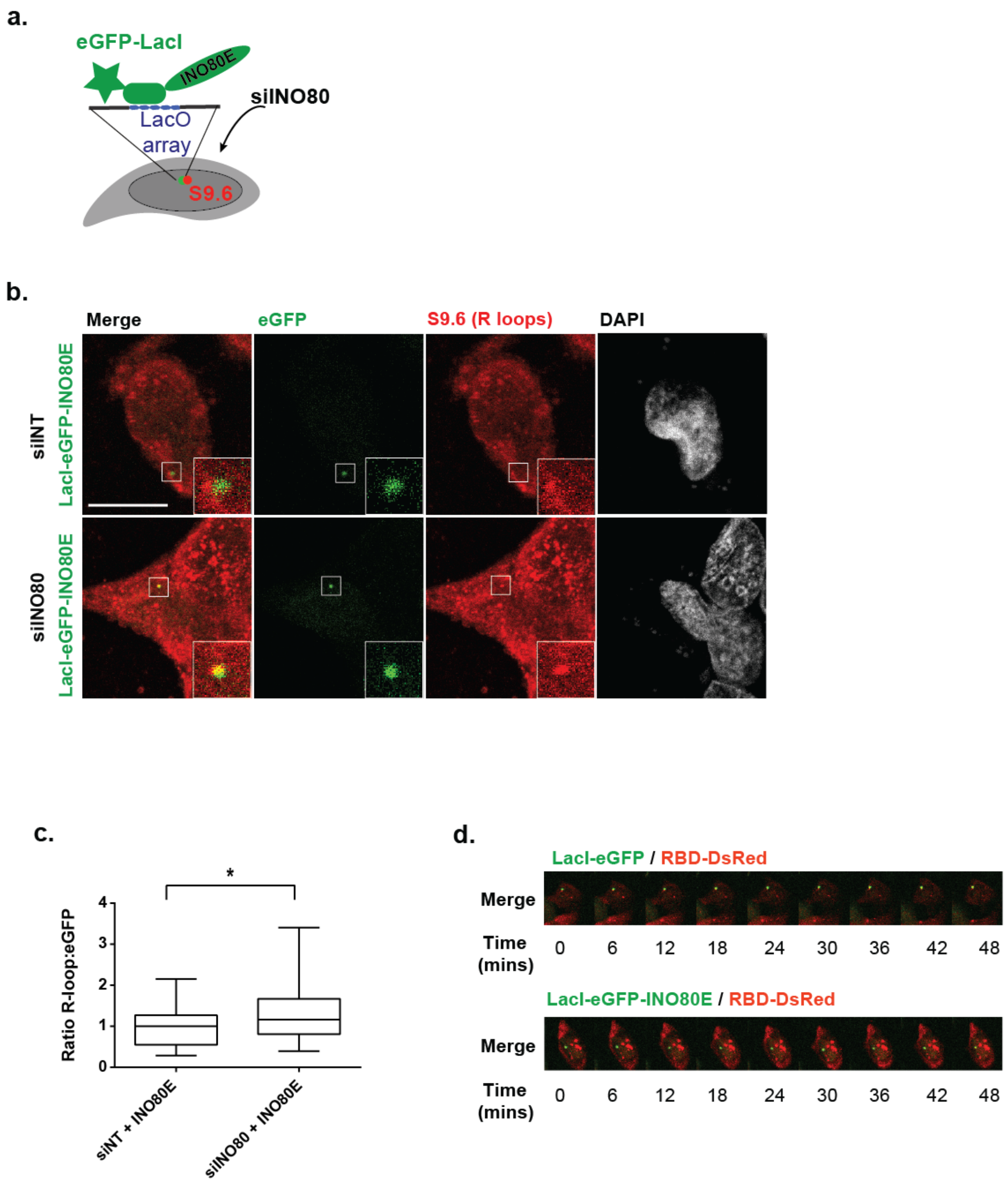

Supplementary Figure 11. R-loops at LacO array.

(a) Schematic representation of the experimental assay. U2OS cells harbouring a 256x LacO array were co-transfected with either control LacI-EGFP or LacI-eGFP-INO80E plasmids and esiRNAs against GFP (siNT) or INO80 (siINO80). Immunostaining with S9.6 after 24 hrs allows visualization of R-Loops at the LacO locus. **(b)** Representative immunofluorescent images of LacO cells transfected with eGFP-LacI and eGFP-LacI-INO80E. Scale bar is 10µm. **(c)** Quantification of the S9.6 signal intensity relative to the underlying eGFP-LacI-INO80E intensity at the LacO locus in siGFP and siINO80 cells. Plot shown is min to max values with line at median. N=3 experiments *p-value =0.0166, (two-tailed unpaired Student's t-test) . **(d)** Selected images of whole cells from Figure 7f are shown from a representative live imaging experiment. Merge shows LacO co-localisation with LacI-eGFP or LacI-INO80E cell during 48 minutes of the time course analysis for RBD-DsRed and LacI-tagged proteins.

Supplementary Figure 12

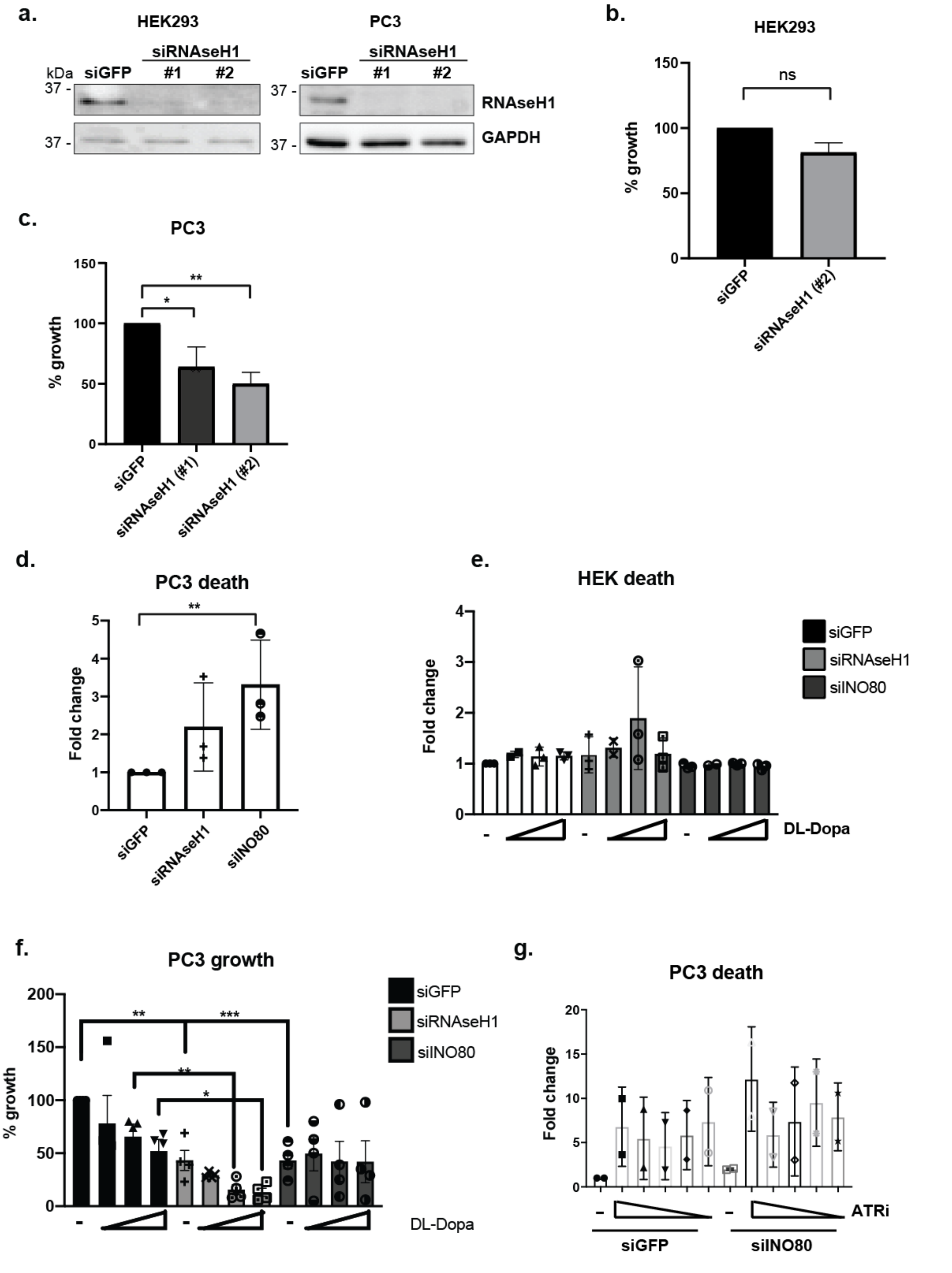

Supplementary Fig. 12. Chemogenetic interactions of INO80 and RNAseH1 in cell death response.

(a) Representative image of RNAse H1 knockdown. HEK293 (left panel) and PC3 (right panel) cells were transfected with two different siRNAs against RNAse H1 (#1 and #2). Western blot was conducted from total extracts with an antibody against RNAse H1 3 days after transfection. **(b, c)** Cells from the HEK293 (b) and PC3 (c) lines were transfected with control (siGFP) or RNAse H1 (siRNAseH1) siRNAs. Growth was analysed as in Figure 8. Data are from 3 independent experiments. siGFP v siRNAseH1#1 *p-value =0.0206, siGFP v siRNAseH1#2 **p-value =0.0010 **(d)** Cell death analysis. PC3 cells transfected with control (siGFP), RNase H1-targeting (siRNaseH1) or INO80-targeting (siINO80) siRNAs were analysed for cell growth and cytotoxicity as in Figure 8e. siGFP v siINO80**p-value =0.0036 **(e)** HEK293 cells were analysed for cell growth and cytotoxicity in the absence or presence of DL-Dopa as in Figure 8e. Data are from 3 independent experiments. **(f)** PC3 cells transfected with control (siGFP), RNase H1-targeting (siRNaseH1) or INO80-targeting (siINO80) siRNAs for 24 hours were treated with increasing concentrations of DL-Dopa for further 7 days incubation and analysed for cell growth as in Figure 8. siGFP v siRNAseH1 **p-value=0.001, siGFP v siINO80 ***p-value =0.0003, siGFP 2uM v siRNAseH1 2uM **p-value =0.0078, siGFP 5uM v siRNAseH1 5uM *p-value =0.0169 **(g)** PC3 cells transfected with control (siGFP), or INO80-targeting (siINO80) siRNAs for 24 hours were treated with increasing concentrations of ATR inhibitor (ATRi) for further 7 days incubation and analysed as in Figure 8e. ns, non-significant; *p-value < 0.05; **p-value < 0.01, ***p-value < 0.005; two-tailed unpaired Student's t-test. Source data are provided as a Source Data file.

Supplementary Table 1.

	Channel 1	v	Channel 2	Pearson	CCFmax Original Image	CCFmin Shifted Image
Cell 1	S9.6	v	INO80	0.082	0.082	-0.005
	EdU	v	INO80	0.009	0.015	0.009
	EdU	v	S9.6	-0.003	0.002	-0.003
Cell 2	S9.6	v	INO80	0.11	0.114	0.012
	EdU	v	INO80	-0.023	-0.008	-0.027
	EdU	v	S9.6	-0.003	0.025	-0.01
Cell 3	S9.6	v	INO80	0.121	0.124	0.048
	EdU	v	INO80	0.047	0.053	0.044
	EdU	v	S9.6	-0.047	-0.033	-0.049
Cell 4	S9.6	v	INO80	0.314	0.314	0.254
	EdU	v	INO80	-0.075	-0.07	-0.077
	EdU	v	S9.6	-0.158	-0.152	-0.159

A Pearson correlation co-efficient was calculated for the co-localisation between each channel in the image. CFF max value is the calculated Pearson correlation co-efficient Channel 1 with the original image of Channel 2. The CCF min value is the calculated Pearson correlation co-efficient of Channel 1 compared to a version of Channel 2 which has been shifted in orientation. If CCF max and min are equal, then the co-localisation observed between the two channels is random. If the CCF max is larger or smaller than the CCF min the co-localisation is non-random or true co-localisation. The Pearson value for the co-localisation between the S9.6 and INO80 channels in Cell 1 is 0.082. When the S9.6 signal is compared to a shifted version of Channel 2(INO80) signal, the Pearson value is -.005.

1 Fox, M. H. A model for the computer analysis of synchronous DNA distributions obtained by flow cytometry. *Cytometry* **1**, 71-77, doi:10.1002/cyto.990010114 (1980).
