# Supplementary material for: Resolution of R-loops by INO80 promotes DNA replication and maintains cancer cell proliferation and viability
Source: Nat Commun. 2020 Sep 10;11:4534. doi: 10.1038/s41467-020-18306-x (PMC7484789; doi:10.1038/s41467-020-18306-x)
Supplement: Supplementary file 3 — Description of Additional Supplementary Files [file 41467_2020_18306_MOESM3_ESM.pdf]

Description of Additional Supplementary Files

File Name: Supplementary Movie 1a & 1b
Description: LacO-carrying U2OS cells were co-transfected with RBD-DsRed and LacI-eGFP plasmids and live cell imaging carried out in Z stacks imaged every 6 minutes. Supplementary Movie 1b shows the RBD-DsRed alone.

File Name: Supplementary Movie 2a & 2b
Description: LacO-carrying U2OS cells were co-transfected with RBD-DsRed and LacI-eGFP-INO80E plasmids and live cell imaging carried out in Z stacks imaged every 6 minutes. Supplementary Movie 2b shows the RBD-DsRed alone.
