# Supplementary material for: Resolution of R-loops by INO80 promotes DNA replication and maintains cancer cell proliferation and viability
Source: Nat Commun. 2020 Sep 10;11:4534. doi: 10.1038/s41467-020-18306-x (PMC7484789; doi:10.1038/s41467-020-18306-x)
Supplement: Supplementary file 8 — Reporting Summary [file 41467_2020_18306_MOESM8_ESM.pdf]

nature research

Corresponding author(s): Anastas Gospodinov and Manolis Papamichos-Chronakis

Last updated by author(s): Sep 5, 2020

# Reporting Summary

Nature Research wishes to improve the reproducibility of the work that we publish. This form provides structure for consistency and transparency in reporting. For further information on Nature Research policies, see our Editorial Policies and the Editorial Policy Checklist.

## Statistics

For all statistical analyses, confirm that the following items are present in the figure legend, table legend, main text, or Methods section.

| n/a | Confirmed | |
|---|---|---|
| ☐ | ☒ | The exact sample size (*n*) for each experimental group/condition, given as a discrete number and unit of measurement |
| ☐ | ☒ | A statement on whether measurements were taken from distinct samples or whether the same sample was measured repeatedly |
| ☐ | ☒ | The statistical test(s) used AND whether they are one- or two-sided<br>*Only common tests should be described solely by name; describe more complex techniques in the Methods section.* |
| ☐ | ☒ | A description of all covariates tested |
| ☐ | ☒ | A description of any assumptions or corrections, such as tests of normality and adjustment for multiple comparisons |
| ☐ | ☒ | A full description of the statistical parameters including central tendency (e.g. means) or other basic estimates (e.g. regression coefficient) AND variation (e.g. standard deviation) or associated estimates of uncertainty (e.g. confidence intervals) |
| ☐ | ☒ | For null hypothesis testing, the test statistic (e.g. *F*, *t*, *r*) with confidence intervals, effect sizes, degrees of freedom and *P* value noted<br>*Give P values as exact values whenever suitable.* |
| ☒ | ☐ | For Bayesian analysis, information on the choice of priors and Markov chain Monte Carlo settings |
| ☐ | ☒ | For hierarchical and complex designs, identification of the appropriate level for tests and full reporting of outcomes |
| ☐ | ☒ | Estimates of effect sizes (e.g. Cohen's *d*, Pearson's *r*), indicating how they were calculated |

*Our web collection on statistics for biologists contains articles on many of the points above.*

## Software and code

Policy information about availability of computer code

| | |
|---|---|
| Data collection | Image acquisition software: Leica Application Suite X (LAS X) (Leica); AxioVision v4.8.1 (Carl Zeiss); Syngene GeneSys v1.7.1; LI-COR Image Studio;Andor Fusion software<br>Previously processed public ChIPseq was downloaded from http://epistemnet.bioinfo.cnio.es/download/ and new datasets were downloaded from ArrayExpress (INO80, E-GEOD-49137) and NCBI GEO (DRIPseq, GSE67581) |
| Data analysis | Image analysis:FIJI/Image J V1.52p; Imaris 9.0 ; SVI Huygens 18.04 (www.svi.nl); CellProfiler; GraphPad Prism v8; BEDTools v2.25.087; Bioconductor's package TxDb.Mmusculus.UCSC.mm9.knownGene (version 3.2.2);ChromHMM https://github.com/EpiStemNet. For ChIPseq analysis have re-used the code available at https://github.com/EpiStemNet/ |

For manuscripts utilizing custom algorithms or software that are central to the research but not yet described in published literature, software must be made available to editors and reviewers. We strongly encourage code deposition in a community repository (e.g. GitHub). See the Nature Research guidelines for submitting code & software for further information.

## Data

Policy information about availability of data

All manuscripts must include a data availability statement. This statement should provide the following information, where applicable:

- Accession codes, unique identifiers, or web links for publicly available datasets
- A list of figures that have associated raw data
- A description of any restrictions on data availability

The authors declare that the data supporting the findings of this study are available within the paper and its Supplementary information files. The datasets generated during and/or analysed during the current study are available from the corresponding author on reasonable request. Raw data files for western blots in all figures are contained in the Raw Data file.

Espresso Database:https://www.ebi.ac.uk/teichmann-srv/espresso INO80 and DRIP ChIPseq data URLs:
https://www.ebi.ac.uk/arrayexpress/experiments/E-GEOD-49137/
https://www.ncbi.nlm.nih.gov/geo/query/acc.cgi?acc=GSE67581
Chromatin State data URL:
http://epistemnet.bioinfo.cnio.es/download/

# Field-specific reporting

Please select the one below that is the best fit for your research. If you are not sure, read the appropriate sections before making your selection.

☒ Life sciences ☐ Behavioural & social sciences ☐ Ecological, evolutionary & environmental sciences

For a reference copy of the document with all sections, see nature.com/documents/nr-reporting-summary-flat.pdf

# Life sciences study design

All studies must disclose on these points even when the disclosure is negative.

**Sample size**

No statistical method was used to predict sample size. The sample size was guided by preliminary studies we carried out, and guided by a number of previous publications using similar approaches. Sample size was kept similar between tested conditions. We collected a minimum of 30 cells for each condition imaged to generate robust statistical data. As the number of cells collected varied depending on the experimental conditions, all cells imaged during any experiment were quantified.
Mijic et al., Nat Commun., DOI: 10.1038/s41467-017-01164-5
Massou, S., Nunes Vicente, F., Wetzel, F. et al. Cell stretching is amplified by active actin remodelling to deform and recruit proteins in mechanosensitive structures. Nat Cell Biol (2020). https://doi.org/10.1038/s41556-020-0548-2

Papers that use similar numbers of cells to analysed H2AX nuclear intensities in response to replication stress are for example:
Mazouzi, A. et al. A Comprehensive Analysis of the Dynamic Response to Aphidicolin-Mediated Replication Stress Uncovers Targets for ATM and ATMIN. Cell Rep 15, 893-908, doi:10.1016/j.celrep.2016.03.077 (2016);
Bianco, J. N. et al. Overexpression of Claspin and Timeless protects cancer cells from replication stress in a checkpoint-independent manner. Nat Commun 10, 910, doi:10.1038/s41467-019-08886-8 (2019);
Fibre labeling analysis of similar number of tracks is suggested and done in the following studies:
Blackford, A. N. et al. The DNA translocase activity of FANCM protects stalled replication forks. Hum Mol Genet 21, 2005-2016, doi:10.1093/hmg/dds013 (2012);
Lemacon, D. et al. MRE11 and EXO1 nucleases degrade reversed forks and elicit MUS81-dependent fork rescue in BRCA2-deficient cells. Nat Commun 8, 860, doi:10.1038/s41467-017-01180-5 (2017);
Schwab, R. A., Nieminuszczy, J., Shin-ya, K. & Niedzwiedz, W. FANCJ couples replication past natural fork barriers with maintenance of chromatin structure. J Cell Biol 201, 33-48, doi:10.1083/jcb.201208009 (2013);
Schwab, R. A. & Niedzwiedz, W. Visualization of DNA replication in the vertebrate model system DT40 using the DNA fiber technique. J Vis Exp, e3255, doi:10.3791/3255 (2011).

**Data exclusions**

No data were excluded.

**Replication**

All relevant experiments were completed a minimum of three times, unless stated otherwise. All repeats were successful, error bars reflect the variation between experiments.

**Randomization**

Experiments were not randomized. Individual cells/DNA fibres/samples in experiments were analyzed in the same way.

**Blinding**

Samples were assigned numbers instead of labels and quantification of images was performed by different researcher than the one who acquired the images. Individual cells/DNA fibres/samples in experiments were analyzed in the same way.

# Reporting for specific materials, systems and methods

We require information from authors about some types of materials, experimental systems and methods used in many studies. Here, indicate whether each material, system or method listed is relevant to your study. If you are not sure if a list item applies to your research, read the appropriate section before selecting a response.

## Materials & experimental systems

| n/a | Involved in the study | |
|---|---|---|
| ☐ | ☒ | Antibodies |
| ☐ | ☒ | Eukaryotic cell lines |
| ☒ | ☐ | Palaeontology and archaeology |
| ☒ | ☐ | Animals and other organisms |
| ☒ | ☐ | Human research participants |
| ☒ | ☐ | Clinical data |
| ☒ | ☐ | Dual use research of concern |

## Methods

| n/a | Involved in the study | |
|---|---|---|
| ☐ | ☒ | ChIP-seq |
| ☐ | ☒ | Flow cytometry |
| ☒ | ☐ | MRI-based neuroimaging |

## Antibodies

Antibodies used

anti-INO80, Proteintech 18810-1-AP
anti-INO80, Abcam ab118787
S9.6 custom made from S9.6 hybridoma, ATCC® HB-8730
anti-RNAse H1, Invitrogen PA5-30974
anti-GAPDH, Santa Cruz Biotechnology 6C5
anti-Tubulin, Santa Cruz sc-5286
anti-Histone H3, Merck Millipore 06-755
anti-phosphoH2AX (Ser 139), clone 2F3, BioLegend, 613401, lots: B219075, B219074
anti-BrdU, clone B44, Becton Dickinson, 347580, lots: 9172603, 7157935
anti-BrdU, Abcam, ab6326, lot: GR3289293-3
anti-actin, Abcam, ab8226, lot: GR3299142-1
anti-ssDNA, Millipore MAB3034, lot: 3209139
anti-pChk1(S345), Cell Signaling 2341T, lot: 8
anti Myc-tag, clone 9B11, Cell Signaling 2276S, lot: 24
anti-mouse IgG DyLight488, Abcam, #ab96879, lot: GR252791-1
anti-rat IgG DyLight594, Abcam #ab96889, lot: GR263830-2
anti-rabbit IgG DyLight594, Abcam #ab96873
anti-mouse IgG AlexaFluor647, Molecular probes #A-31571
AffiniPure Donkey Anti-mouse IgG AlexaFluor647, Invitrogen A-31571
AffiniPure Donkey Anti-Rabbit IgG Alexa Fluor 594 Invitrogen A-32740
anti-rabbit IRDye680, Li-Cor,#926-32221
anti-mouse IRDye800CW, Li-Cor, #926-32210

Validation

Validation data is provided by each manufacturer online, summarised below. In addition, we validated antibodies by using knockdown controls as described in the manuscript. anti-BrdU, clone B44, and Rat anti-BrdU - were also validated by fibre labelling omitting the appropriate label. S9.6 -validated by RNAseH1 overexpression and in vitro treatment of samples with recombinant RNAseH as described in the manuscript. anti-ssDNA - validated by ELISA and byproducing correct signal overlapping CldU and IdU signal there anti-pChk1(S345) - validated by Western anti-INO80 -validated by knockdown anti-RNAse H1 -validated by IHC (P) and Western anti-H3 -validated by ChIP, ICC & Western; anti-GAPDH -validated by Western anti Myc-tag, clone 9B11 - verified by Western.

Validation statements of primary antibodies on manufacturer's websites and applications:

anti-phosphoH2AX (Ser 139), clone 2F3, - manufacturer's website and citations therein: https://www.biolegend.com/en-us/products/purified-anti-h2a-x-phospho-ser139-antibody-1990

anti-BrdU, clone B44, product datasheet: https://www.bdbiosciences.com/ds/is/tds/23-1349.pdf,
anti-BrdU, Abcam, ab6326, – product datasheet, https://www.abcam.com/brdu-antibody-bu175-icr1-proliferation-marker-ab6326.html,
anti-ssDNA – antibody datasheet: https://www.merckmillipore.com/INTL/en/product/Anti-DNA-Antibody-single-stranded-clone-16-19,MM_NF-MAB3034?ReferrerURL=https%3A%2F%2Fwww.google.com%2F&bd=1,
Application of the anti-BrdU antibodies and ssDNA antibody in DNA fibre labelling:
Quinet, A., Carvajal-Maldonado, D., Lemacon, D. & Vindigni, A. DNA Fiber Analysis: Mind the Gap! Methods Enzymol 591, 55-82, doi:10.1016/bs.mie.2017.03.019 (2017);
Schwab, R. A. & Niedzwiedz, W. Visualization of DNA replication in the vertebrate model system DT40 using the DNA fiber technique. J Vis Exp, e3255, doi:10.3791/3255 (2011).

## Eukaryotic cell lines

Policy information about cell lines

Cell line source(s)

U2OS-lacO-IScel-Tet19 cell line was a gift from Dr. Soutoglou (IGBMC, FRANCE). PC3 cells, HEK293, WM1361, MCF7 were sourced from the Newcastle University Biobank.

Authentication

All cell lines were authenticated prior to the commencement of work using short tandem repeat profiling (LGC Standards)

Mycoplasma contamination

Cell lines tested negative for mycoplasma.

Commonly misidentified lines
(See ICLAC register)

No commonly misidentified cell lines were used in the study

# ChIP-seq

## Data deposition

☐ Confirm that both raw and final processed data have been deposited in a public database such as GEO.

☐ Confirm that you have deposited or provided access to graph files (e.g. BED files) for the called peaks.

| | |
|---|---|
| Data access links<br>*May remain private before publication.* | *For "Initial submission" or "Revised version" documents, provide reviewer access links. For your "Final submission" document, provide a link to the deposited data.* |
| Files in database submission | *Provide a list of all files available in the database submission.* |
| Genome browser session<br>(e.g. UCSC) | *Provide a link to an anonymized genome browser session for "Initial submission" and "Revised version" documents only, to enable peer review. Write "no longer applicable" for "Final submission" documents.* |

## Methodology

| | |
|---|---|
| Replicates | *Describe the experimental replicates, specifying number, type and replicate agreement.* |
| Sequencing depth | *Describe the sequencing depth for each experiment, providing the total number of reads, uniquely mapped reads, length of reads and whether they were paired- or single-end.* |
| Antibodies | *Describe the antibodies used for the ChIP-seq experiments; as applicable, provide supplier name, catalog number, clone name, and lot number.* |
| Peak calling parameters | *Specify the command line program and parameters used for read mapping and peak calling, including the ChIP, control and index files used.* |
| Data quality | *Describe the methods used to ensure data quality in full detail, including how many peaks are at FDR 5% and above 5-fold enrichment.* |
| Software | *Describe the software used to collect and analyze the ChIP-seq data. For custom code that has been deposited into a community repository, provide accession details.* |

# Flow Cytometry

## Plots

Confirm that:

☒ The axis labels state the marker and fluorochrome used (e.g. CD4-FITC).

☒ The axis scales are clearly visible. Include numbers along axes only for bottom left plot of group (a 'group' is an analysis of identical markers).

☒ All plots are contour plots with outliers or pseudocolor plots.

☒ A numerical value for number of cells or percentage (with statistics) is provided.

## Methodology

| | |
|---|---|
| Sample preparation | Cells were collected by trypsinization, collecting cell medium as well. Following centrifugation and washes they were fixed in 70% ethanol for at least a day at -20 deg C. After washes and rehydration, RNA was digested with RNase A (1h, 37 deg C) and stained with propidium iodide |
| Instrument | Becton Dickinson Facscalibur |
| Software | Software: Acquisition - CellQuest, Analysis - FlowJo v. 8.7 |
| Cell population abundance | No cells were excluded during acquisition, at least 10000 events were counted per sample. |
| Gating strategy | Only debris were excluded using gating as shown on supp. fig 3b. Less than 10% of population was excluded. |

☒ Tick this box to confirm that a figure exemplifying the gating strategy is provided in the Supplementary Information.
